# Information disclosure, practical actions and dynamics of employees' health and safety issues in Chinese family businesses—Evidence based on Chinese a-share listed companies

**DOI:** 10.3389/fpubh.2022.952823

**Published:** 2022-07-22

**Authors:** Yi Guo, Aijun Li, Yi Zhou, Yuna Di

**Affiliations:** ^1^School of International Economics and Management, Beijing Technology and Business University, Beijing, China; ^2^School of Economics, Beijing Technology and Business University, Beijing, China

**Keywords:** health and safety, family firms, occupational health, employee health, corporate sector, corporate social responsibility, family business, game theory

## Abstract

**Introduction:**

Global health emergency as COVID-19 has brought unprecedented concerns to the health and safety of employees, which is important yet long-neglected. This paper studies the mechanism and influencing factors of Chinese family enterprises performance in employees' health and safety from information disclosure, practical action and dynamic change. And based on theoretical framework and empirical model, this paper provides feasible regulatory policies on the behavior of family business.

**Methods:**

This study construct a game theory framework and uses a sample of Chinese A-share listed companies. The database is provided by a third-party corporate social responsibility rating agency, SynTao Green Finance. We use empirical models to test the hypothesis from the theoretical model of game theory.

**Results:**

In practice, family businesses are less likely to fulfill the health and safety responsibilities of employees compared to non-family businesses. Family businesses are likely to be more motivated than other businesses to send signals that they are performing their responsibilities well. From the view of operation term, family businesses will be gradually inclined to better fulfill the health and safety responsibilities of their employees, while this process will show a “U” shape change over operation time.

**Conclusions:**

As there is inconsistency between the information disclosure and actual practice of family enterprises when it comes to the issue of employee health and safety, more related regulatory policies and stakeholder monitoring are needed. Although the performance of family enterprises in this regard will be better in the long run, it is still necessary to improve employees' legal and rights awareness and enhance the effectiveness of supervision over external stakeholders.

## Introduction

The impact of COVID-19 has brought unprecedented attention to the health and safety of employees around the world. The so-called family business, a long-established and ubiquitous business model worldwide, is considered to play an important role in the economic ecology of almost all countries (1). As stated by some reports, family businesses contribute 70–90% of global GDP (2). In China, the number of family businesses is also on a rapid rise in China, and they have accounted for ~80% of Chinese private companies. However, there is few research on the health and safety of employees in family businesses. Although family businesses are the most common operating mode in private enterprises, the availability of information about family businesses in China is limited compared with other private enterprises. Generally, it is easier to obtain the relevant information of listed companies, whereas there are only about 1,000 family-owned companies (the number varies as standard changes) among the more than 3,000 private listed companies in China's A-share market. This feature not only brings the challenge of lack of available information, cases and data to the relevant research, but also makes it more difficult for people to understand the occupational health and safety status of employees in family business under the condition of information asymmetry, let alone to discuss strategies and policies for improvement in their welfare. Therefore, we need proper theory framework and solid data to analyze the behavior of Chinese family business in employees' health and safety issues.

Based on the above reality, the innovation of this paper is reflected in both theory and practice. In theory, we attempt to build an analytical framework for the health and safety issues of family business employees, which are less studied. In practice, this study focuses on the performance of family businesses in China's social environment, and attempts to provide potential policies on external supervision from stakeholders. The contributions of this study are as follows: First, this study unifies the existing theoretical attempts on corporate fulfillment of employee health and safety responsibilities, and provides a certain amount of evidence for the solution of the existing disputes. Second, From the perspective of actual actions and signals released by family enterprises, this paper explains the impact of family enterprises on employees' performance of health and safety responsibilities, and attempts to reveal the practical motivation of family enterprises to perform employees' health and safety responsibilities. Third, in the case of difficult access to information and data, this paper applies limited information and data to take into account the possible changes in the time dimension of family enterprises' implementation of employee health and safety behaviors, and combine with the traditional Chinese cultural environment to explain the possible reasons behind such behavioral changes in Chinese family enterprises, providing inspiration for future related research. The conclusions are conducive to vulnerable stakeholders and external stakeholders when encountering irresponsibility in the course of getting along with family enterprises. The actions taken in-between lays a foundation to some extent, which will play a role in promoting family businesses to improve the level of environmental responsibility and improve the relevant information disclosure system of the listed family business company.

## Literature review

At present, the most common theories for analyzing corporate social responsibility (CSR) mainly include agency theory and social emotional wealth theory (socio-emotional wealth, SEW) (3).

From the perspective of agency theory, the controller of a family business, as a rational participant in the market, naturally faces the problem of obtaining maximum benefit with limited resources (4). Existence also brings “principal-agent” problems (5). When family businesses are faced with two types of “principal-agent” problems, the “first type” could generally be solved because their family members are widely involved in specific operations as senior managers. However, as the family members' voices increase, they have more incentives and conditions to encroach on the interests of other stakeholders (6), which makes it possible for the holding family to reduce costs and seek private interests while ignoring the health and safety of employees. In non-family businesses, major shareholders do not have such a powerful voice compared to other stakeholders. This view is supported by studies by Abdullah et al. (7). In addition, considering that many enterprises are state-owned enterprises in China in practice, these enterprises are not completely in seek of self-interest, and are inclined to take on more such social responsibility as those for the health and safety of employees. Therefore, family businesses have the tendency to take up less responsibility for the health and safety of their employees.

However, the socio-emotional wealth theory represented by such scholars as Gomez-Mejia takes into consideration the influence of family management on enterprise management decision-making, which will cause enterprises to seek non-economic goals such as socio-emotional wealth in addition to maximizing their own economic interests (8). What can also be seen as part of this socio-emotional wealth is the family image and reputation built by shouldering more responsible for the health and safety of employees, and the moral capital accumulated. These motivations may incentivize family firms to pursue certain non-economic interests, thereby accumulating more socio-emotional wealth for the family. In non-family businesses, however, this motivation may be weakened by the belief that stakeholders are more dispersed, reducing the motivation to acquire social-emotional wealth, which was supported by the research of Jiang et al. (9). In addition, as shown by the research results of Chen et al. (10) on Chinese enterprises, such factors as organizational image and reputation will exert an influence on Chinese enterprises' performance of their social responsibilities.

In fact, the above two seemingly contradictory theories are not tit for tat. The cost problems caused by enterprises taking responsibility for employees' health and safety under the consideration of principal-agent theory has mostly been rooted within the firm, which is mainly the result of the game between family firms and their employees. However, the social emotional wealth emphasized by the social emotional wealth theory fundamentally derives from the external stakeholders of the firm.

The main shareholders and senior managers of family firms are frequently closely related. On the one hand, they are in a stronger position for such internal stakeholders as employees than in general firms; On the other hand, they also have greater advantages of information asymmetry over the external stakeholders of the firm (government, community, etc.). Therefore, family businesses have greater possibility to ignore employees' health and safety issues in practice, while relying on asymmetric advantages to send a responsible signal to their external stakeholders to seek social emotional wealth. Preuss and Lenssen (11) and Prior et al. (12) illustrates the possibility of this type of motivation from one side.

On this basis, we established the following analytical framework.

**Figure 1:**
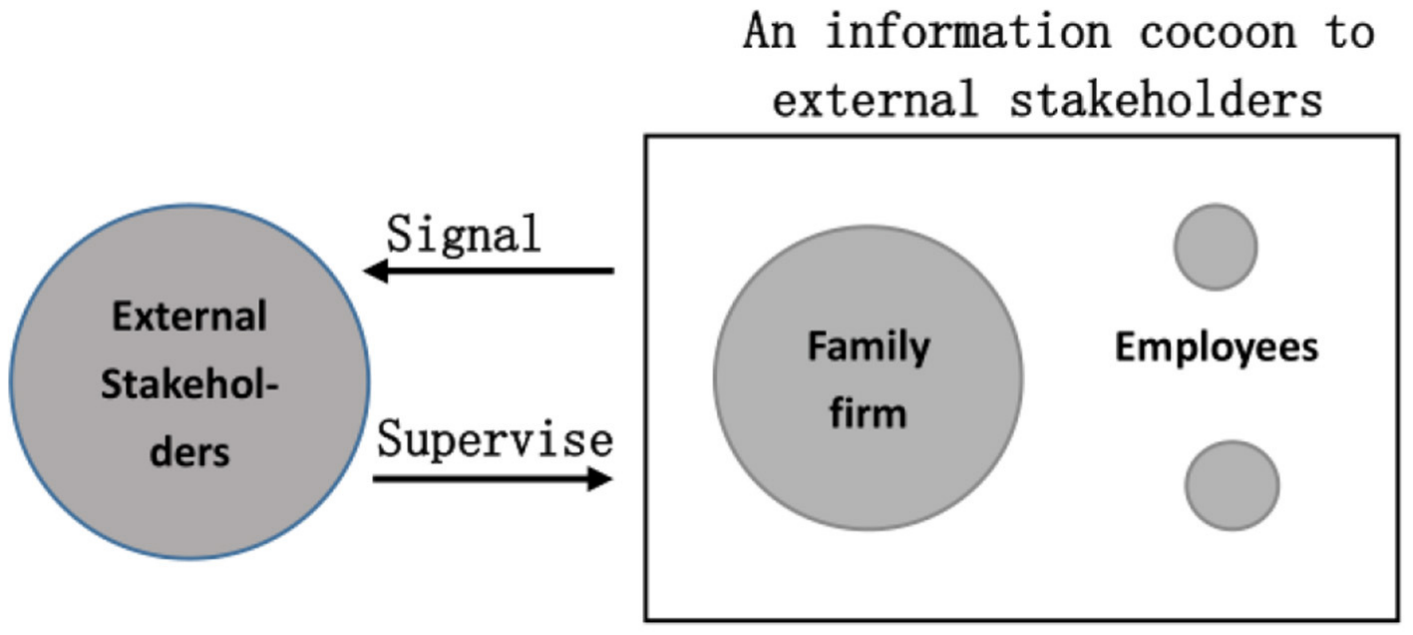
Analysis framework.

As shown in Figure 1, the family business engages in a game with relatively weak employees, ultimately resulting in the outcome of whether and to what extent it is responsible for employees' health and safety. External stakeholders such as the government will accept the signals from the firm and supervise the family firm. However, the main shareholders and senior managers of family enterprises often come from the same family, therefore there is interest binding, and a greater possibility of collusion as well. Therefore, the family business enjoys a greater information advantage for its own information and internal behavior. At the time, compared to other enterprises, their external signals and supervision tend to be ineffective.

This behavior pattern of family businesses is also supported by the literature. Nekhili et al. (13)'s research on French family businesses shows that the disclosure of social responsibility information of family businesses is less than that of non-family businesses. And the Tobin Q of family enterprises is positively correlated with their social responsibility disclosure, which shows that family enterprises can indeed obtain social emotional wealth by disclosing social responsibility information to a certain extent. Izzo and Ciaburri (14)'s research shows that family businesses do take social responsibility actions selectively according to changes in the social environment, which also indirectly shows that family businesses have strategic choices in fulfilling their social responsibilities.

Based on this analytical framework, the model established by Wang on CSR in China is improved to make it more relevant to the specific context of the issues discussed above, with the aim of setting up a game theory model for employee health and safety issues between family firms and their employees and external stakeholders (15).

## Methods and data

### Game theory modeling analysis

#### Game model of family businesses and employees on employee occupational health and safety

In the game between employees and enterprises in terms of employees' occupational health and safety, the payment matrix is considered as follows.

As shown in Table 1, companies and employees have two sets of action plans for employees' health and safety game. A represents a relatively strong/positive approach, while B represents a relatively weak/negative approach. *a*_1_ and *a*_2_ in the matrix signify the gains and losses of employees and enterprises when equilibrium is not reached, respectively. To simplify the analysis, it is assumed that both are less than zero, which is equivalent to assuming that it will be a lose-lose outcome if the equilibrium cannot be reached. However, it is worth noting that the two sides of the game have different opinions regarding employee health and safety issues. *b*_1_ and *b*_2_ indicate the additional benefits that employees and firms receive contributed by their own strong strategies and the other's weak strategies when the equilibrium is achieved, and *b*_1_, *b*_2_ and k are all greater than zero. Compared with employees, companies, especially family-owned companies, own stronger negotiating power, so there is *a*_1_ < *a*_2_, *b*_1_ < *b*_2_.

**Table 1:** Payment matrix between employees and firms.

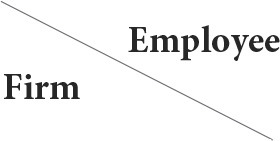	**A**	**B**
A	*a*_1_, *a*_2_	*k, k* + *b*_2_
B	*k* + *b*_1_, *k*	*a*_1_, *a*_2_

By considering the incomplete information mixed strategy equilibrium (MNE) of this game, what is pinpointed is the probability that the enterprise and the employee adopt two strategies respectively, and use this probability to judge the inclination of the two sides of the game to adopt the two strategies. It is assumed that the probability of the enterprise taking action A is P, the probability of taking action B is (1-P), the employee's strategy for taking action A is Q, and the strategy for taking action B is (1-Q). From this assumption, we can obtain the trade-off equations for enterprises and employees:


(1) $$\begin{array}{l} a_{2}\times Q\;+\;\left(k\;+\;b_{2}\right)\times \left(1\;-\;Q\right)\;=\;k\times Q\;+\;a_{2}\times \left(1\;-\;Q\right) \end{array}$$

(2) $$a_{1}\times P\;+\;(k\;+\;b_{1})\times (1-P)\;=\;k\times P\;+\;a_{1}\times (1\;-P)$$

Solving the above two equations are shown as follows:


(3) $$P=\;\frac{(k\;+\;b_{1}-\;a_{1})}{(2k+\;b_{1}-\;2a_{1})}$$

(4) $$Q=\;\frac{(k\;+\;b_{2}-\;a_{2})}{(2k+\;b_{2}-\;2a_{2})}$$

The relationship between the size of the following formula and the sizes of *a* and *b* is demonstrated as follows:


(5) $$P=\;\frac{(k\;+\;b-\;a)}{(2k+\;b-\;2a)}$$

The partial derivative of *p* with respect to *a* is illustrated as follows:


(6) $$\frac{\partial p}{\partial a}\;=\;\frac{b}{{(2k+\;b-2a)}^{2}}>0$$

The partial derivative of *p* with respect to *b* is shown as follows:


(7) $$\frac{\partial p}{\partial b}\;=\;\frac{k-a}{{(2k+\;b-2a)}^{2}}>0$$

Because *a*_1_ < *a*_2_ and *b*_1_ < *b*_2_, the company as a strong side is more inclined to take strong actions on employee safety and health issues than the employee side does, and employees are more likely to adopt negative strategies. In other words, it is easier to achieve the pure strategy equilibrium of (*k, k* + *b*_2_) in reality; to put it another way, the enterprise is not responsible for the health and safety of employees, but the employees can only accept the result. Compared with other companies, family businesses have more say in the game of employees; therefore, this kind of equilibrium is more likely to occur in family businesses.

Through an analysis of this part of the model, an assumption is proposed as follows:

**H1:** Compared with other businesses, family businesses tend to ignore employee health and safety issues in practical actions in order to save costs.

#### Game model of family business and external stakeholders

In terms of the game between enterprises and external stakeholders regarding the health and safety of employees, what is considered here in this study is a simple static game of incomplete information. It is assumed that a company is in the course of weighing how well it fulfills its employees' health and safety responsibilities in order to capture social-emotional wealth from external stakeholders. Supposing that the company decides not to perform this obligation well, there is a probability *n* that external stakeholders will detect this behavior and impose penalties on it. Based on this assumption, the possession strategy of a company is set up according to Table 2.

**Table 2:** Payment matrix of corporate dominant strategy.

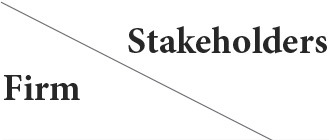	**C**	**D**
Y	f-c	f-c
N	f	f-x

Since the choice made by corporate dominant strategy in this game has nothing to do with the benefits of stakeholders, the benefits of external stakeholders are omitted. As Strategy C of the external stakeholder in the matrix indicates, it has discovered that the company is sending false signals, whereas D indicates that it has done the opposite. If the enterprise fulfills the related responsibilities of employees' occupational health and safety well, it will pay the cost c on the basis of the harvest f, that is, the net benefit at this time is f-c. If firms choose not to perform well, they will pay no cost c if they are not detected; however, they will pay a greater price x than c if they are found.

Calculating the expected return of the two strategies adopted by the enterprise, it can be seen that the expected return of the enterprise choosing to fulfill its responsibility is f-c, and the expected return of not fulfilling its responsibility is f-nx. Obviously, when c is constant, the strategy adopted by the company as dominant strategy depends entirely on the intensity of punishment and the probability of being discovered. When the punishment is the same, the choice of strategy is greatly affected by the probability of being discovered. Starting from the theory in the theoretical research part of this paper, it is clear that family businesses whose interests are highly aligned with major shareholders and senior managers are more motivated to send false signals to the outside world without fulfilling the health and safety responsibilities of their employees. Therefore, there lacks evidence to judge whether the family business has properly fulfilled its employees' health and safety responsibilities through the signals released by the family business. In other words, the signals delivered by family enterprises and non-family enterprises on the issue of employee health and safety are theoretically not significantly different. There will even be cases where family businesses perform better than non-family businesses. Of course, this conclusion is not as strong as that drawn from the game with employees in the previous subsection.

Based on the analysis of this part of the game, assumption could be made as follows:

**H2:** Only relying on the information disclosed to the public, the performance of family enterprises on employee health and safety issues is better than that of non-family enterprises, or there is no significant difference between the two types of enterprises.

#### Complementary to game theoretical models: The time dimension

In the above analysis framework and game model, what is statically discussed is only the behavior of family businesses in fulfilling their employees' health and safety responsibilities, without taking into consideration the impact of changes in the operating stages of family businesses over time. This section will discuss how the responsibility performance behavior of Chinese family businesses will change over time in China's social and cultural environment.

The traditional concept of the family enjoys a long history in China. Family members live in groups with blood ties and have a strong cultural and emotional identity with each other. Under the influence of traditional Chinese culture, a traditional Chinese family will pay greater attention to the improvement of the quality of family members in the process of its development and growth, and will be proud of the prestige and honor it brings from generation to generation. This culture is known in China as Jia-Feng, which refers to the family tradition. In this sense, as the family business continues to grow, the controlling family pays an increasing amount of attention to the inheritance of its family prestige and honor. This is reflected in our analytical framework that firms' propensity to acquire social-emotional wealth increases over time. Li 's (16) research on Chinese family businesses also confirmed this. At the same time, external stakeholders, such as the government, may weaken the unequal position of family businesses in the game through legislation, increase supervision, and provide legal assistance.

However, despite the adjustment of the traditional Chinese cultural influence, what cannot be ignored is the influence of the mechanism discussed above. In addition, the scale of Chinese family enterprises is relatively small compared to other enterprises, which may make them more likely to fall into a crisis of operation or end the operation earlier than expected (17). Employees' health and safety of may be of less importance over time.

Based on this, the following assumption could be made:

**H3:** As the business enjoys a longer history, the family business will better /worse fulfill the health and safety responsibilities of its employees.

Two mechanisms of H3 may play a major role in different periods of family business operations. Thus, assumption could be made as follows:

**H4:** A family business's performance of employee health and safety responsibilities will show a U-shaped or inverted U-shaped change over time.

To confirm these assumptions, the econometric methods below will be applied for demonstration.

### Data and econometric methods

There exists rare domestic literature on family business responsibility for employee health and safety in China. Relevant research data and methods on Chinese CSR in a broader sense are referred to in order to construct our research scheme (18, 19). Because of the difficulty in obtaining data and information about family enterprises mentioned above, most of the domestic CSR studies related to family enterprises have applied the Survey of Private Enterprises in China database in 2012 based on the cross-sectional data obtained from the questionnaire. The indicators related to employee health and safety of Chinese A-share listed companies are conducted by SynTao Green Finance, a third-party CSR rating agency, and the related indicators are from CSMAR database. After excluding financial firms that may behave differently, 3,290 observations were obtained.

All variables used in this study are shown in Tables 3, 4.

**Table 3:** Variable descriptions.

**Variable name**	**Variable meaning**
Prof_tra	SynTao Green Finance scores the enterprise according to the employees' vocational training. The data comes from the “number of employees/times and time of vocational training received by employees each year” in the employee-related indicators of SynTao Green Finance.
H&S_pol	The score given by SynTao Green Finance is based on the company's employee occupational health and safety-related policies published in its annual social responsibility report or other channels.
YoN	Whether the business is a family business or not, different businesses are assigned values according to CSMAR's broad definition of family business, 1 for family businesses and 0 for non-family businesses.
Power	The proportion of the actual controller of the enterprise owning the actual control of the enterprise, the specific value comes from the CSMAR database
Fam_time	The time since the company has been familiarization (years).
SIZE	Business size of family firms, measured by the logarithm of total assets data.
Lev	Enterprise debt ratio family firms, the logarithm of the ratio of its liabilities to total assets.
Rate	Total profit margin of family firms.
Number	Number of board members of family firms.
Indus	Industry fixed effects, refer to the dummy variables assigned to each industry by the CSRC industry classification.

**Table 4:** Data description.

**Variable name**	**Obs**	**Mean**	**Std.Dev**	**Min**	**Max**
Prof_tra	3,290	7.796	17.878	0	100
H&S_pol	3,290	63.016	42.535	0	100
YoN	3,290	0.243	0.429	0	1
Power	1,092	39.575	16.768	0.005	87.920
Fam_time	800	5.068	5.295	0	28
SIZE	787	21.279	0.921	17.053	24.872
Lev	787	−1.083	0.962	−7.861	1.720
Rate	800	0.064	0.069	−0.636	0.664
Number	775	8.002	1.533	4	15

Variable Prof_tra is chosen as the proxy variable of the enterprise's actual action on employee health and safety issue. It is generally believed that good occupational training is conducive to improving the production safety of employees, and that it is a behavior that is responsible for the life and health of employees to conduct good occupational training for employees (20). And it is based on the following considerations: First, professional vocational skills training can make employees improve their proficiency and capability to avoid production hazards, so that employees can produce in a safer manner; Second, the cost of vocational training for employees should be borne by the enterprise; Third, employees cannot produce for the enterprise during the training period, which can be regarded as the opportunity cost shouldered by the enterprise for employees' safety and health. Therefore, the length of occupational training is taken that each employee of the company receives in a year (Prof_tra) as a proxy variable for the company's responsibility for employees' occupational health and safety.

At the same time, we used the variable H&S_pol from SynTao green finance to reflect the performance of employees' health and safety responsibilities disclosed by the enterprise itself. The higher the score, the better the performance of employees' health and safety responsibilities.

Through the distribution of the above two proxy variables, what is intuitively illustrated is the differences in practical actions and information disclosure between family enterprises and non-family enterprises' on employee health and safety issues. In this process, H1 and H2 were tested.

For H3, the following models is established to conduct empirical research on family business observations:


(8) $$\begin{array}{l} Prof\_tra_{i}=\beta_{0}\;+\beta_{1}\;*\;Fam\_time_{1}+\;\beta_{3}\;*control_{i}\\ \;+\beta_{4}\;*Indus_{i}+\;\varepsilon_{i} \end{array}$$

(9) $$\begin{array}{l} H\&S\_pol_{i}=\beta_{0}\;+\beta_{1}\;*\;Fam\_time_{1}+\;\beta_{3}\;*control_{i}\\ \;+\beta_{4}\;*Indus_{i}+\;\varepsilon_{i} \end{array}$$

This part of the study includes only 800 family business observations. Taking the time of family business familiarization as the core explanatory variable, discussion was carried out on the impact of different periods of family business operations on the health and safety responsibilities of traveling employees.

In the actual data processing of this study, the introduction of control variables will inevitably lead to the loss of valuable observations. To avoid the influence of this problem and at the same time ensure the robustness of the empirical results as much as possible, the following two models are set up in the actual processing of each hypothesis for research. The first model relaxes the assumptions by not introducing control variables. The maximum number of observations was maintained, and the relationship between the core explanatory variable and the explained variable was studied. The second model tightens the conditions, introduces control variables, and excludes factors such as state-owned enterprises. Finally, the regression results of the two models are compared. If the core explanatory variables of the two models do not change in sign and significance of the regression coefficients, a relatively robust result could be generated. The regression results are listed in **Table 6**.

At the same time, in order to verify H4, the quadratic term of Fam_time was introduced and the following models was established. The regression results are shown in Model 9–10, and the regression results are presented in **Table 7**.


(10) $$\begin{array}{l} Prof\_tra_{i}=\beta_{0}\;+\beta_{1}\;*\;Fam\_time_{i}+\;\beta_{2}\;*Fam\_time_{i}^{2}\\ \;+\beta_{3}\;*control_{i}+\beta_{4}\;*\;Indus_{i}+\varepsilon_{i} \end{array}$$

(11) $$\begin{array}{l} H\&S\_pol_{i}=\beta_{0}\;+\beta_{1}\;*\;Fam\_time_{i}+\;\beta_{2}\;*Fam\_time_{i}^{2}\\ \;+\beta_{3}\;*control_{i}+\beta_{4}\;*\;Indus_{i}+\varepsilon_{i} \end{array}$$

## Results

### Results of statistical analysis

Table 5 presents the results of the correlation analysis for the variables involved in this study. Among the 3,290 observations here, there were 800 family business samples. In the correlation analysis, it can be intuitively observed as follows: 1. Compared with non-family enterprises, the situation of family enterprises' vocational training for employees is worse, to be specific, in practice, family businesses may be more reluctant to fulfill their employee health and safety responsibilities than non-family businesses, which is consistent with H1; 2. From the disclosure point of view, there is no intuitively significant difference between family businesses and non-family businesses, which is consistent with H2; 3. From the time dimension of corporate familiarization, familiarization time is proportional to its responsibility performance. In summary, the results of the correlation study on variables support H2.

**Table 5:** Correlation matrix of variables.

**Variable**	**Prof_tra**	**H&S_pol**	**YoN**	**Power**	**Fam_time**	**SIZE**	**Lev**	**Rate**	**Number**
Prof_tra	1.000								
H&S_pol	0.107^***^	1.000							
YoN	−0.056^***^	0.008	1.000						
Power	−0.108^***^	0.062^*^	0.173^***^	1.000					
Fam_time	0.252^***^	0.094^***^	Null	−0.291^***^	1.000				
SIZE	0.399^***^	0.164^***^	Null	−0.060	0.449^***^	1.000			
Lev	0.012	0.172^***^	Null	−0.085^**^	−0.170^**^	−0.084^***^	1.000		
Rate	0.042	0.014	Null	0.215^**^	−0.083^**^	0.208^***^	−0.188^***^	1.000	
Number	0.148^***^	0.065^*^	Null	−0.132^***^	−0.136^***^	0.235^**^	0.037	0.066^*^	1.000

*** *Significant at the 1% level*.

** *Significant at the 5% level*.

* *Significant at the 10% level*.

*Variables Prof_tra and H&S_pol are provided by SynTao Green Finance, other variables are sourced from the CSMAR database*.

**Figure 2:**
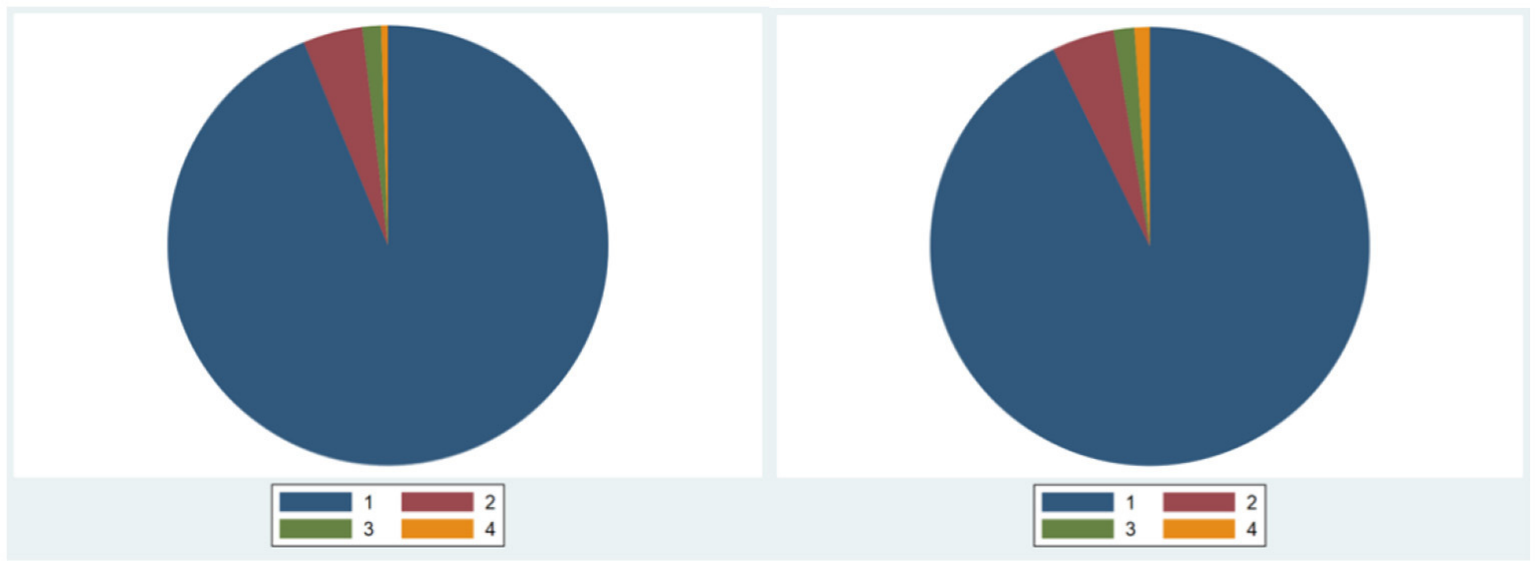
Prof_tra's score distribution.

The sample by family business and non-family business were grouped, and by drawing pie charts for the score intervals of the two variables Prof_tra and H&S_pol for each group, in a more intuitive way, the hypothesis H1 and H2 was initially tested. Prof_tra's score distribution of family and non-family businesses is shown in Figure 2.

**Figure 3:**
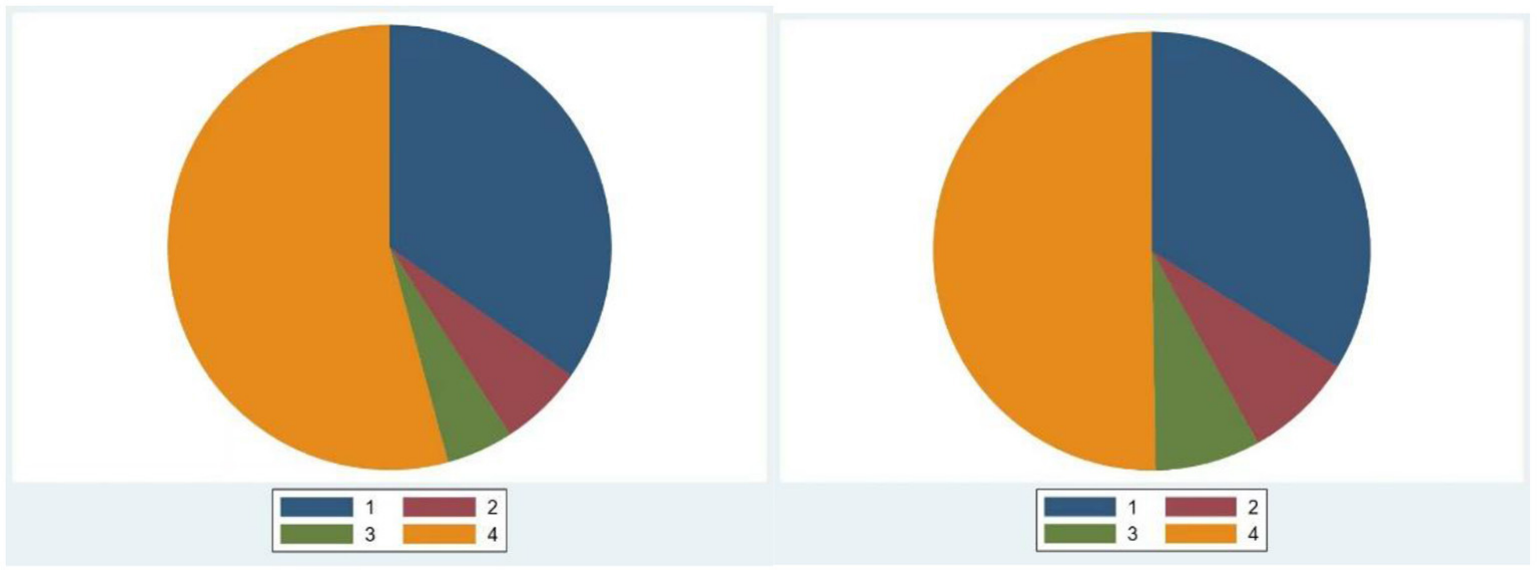
H&S_pol's score distribution.

The different colored areas 1, 2, 3, and 4 in the figure represent the score ranges 0–25, 26–50, 51–75, and 76–100, respectively. The graph on the left represents a family business, and the graph on the right represents a non-family business. In terms of percentages in each interval, non-family businesses score better than family businesses (The percentage of family businesses that fall within the range 1–4 were 93.75, 4.375, 1.375, and 0.5. The corresponding percentages for non-family businesses were 92.77, 4.578, 1.526, and 1.126). This also supports H1 to a certain extent. The distribution of H&S_pol's family and non-family business scores is shown in Figure 3.

The score of family business in H&S_pol is completely different from that in Prof_tra. From the distribution of scores in field H&S_pol, the proportion of family firms in the highest districts is significantly higher than that of non-family firms. In the score distribution of non-family enterprises, the four intervals accounted for 34.75, 6.125, 4.875, and 54.25% respectively. In the score distribution of family enterprises, the four intervals accounted for 33.82, 8.112, 7.791, and 50.28%, respectively. This means that family businesses may have behaviors that are inconsistent with the information disclosed to the outside world and their actual actions, which supports Hypothesis H2 from one aspect.

### Results of regression analysis

**Figure 4:**
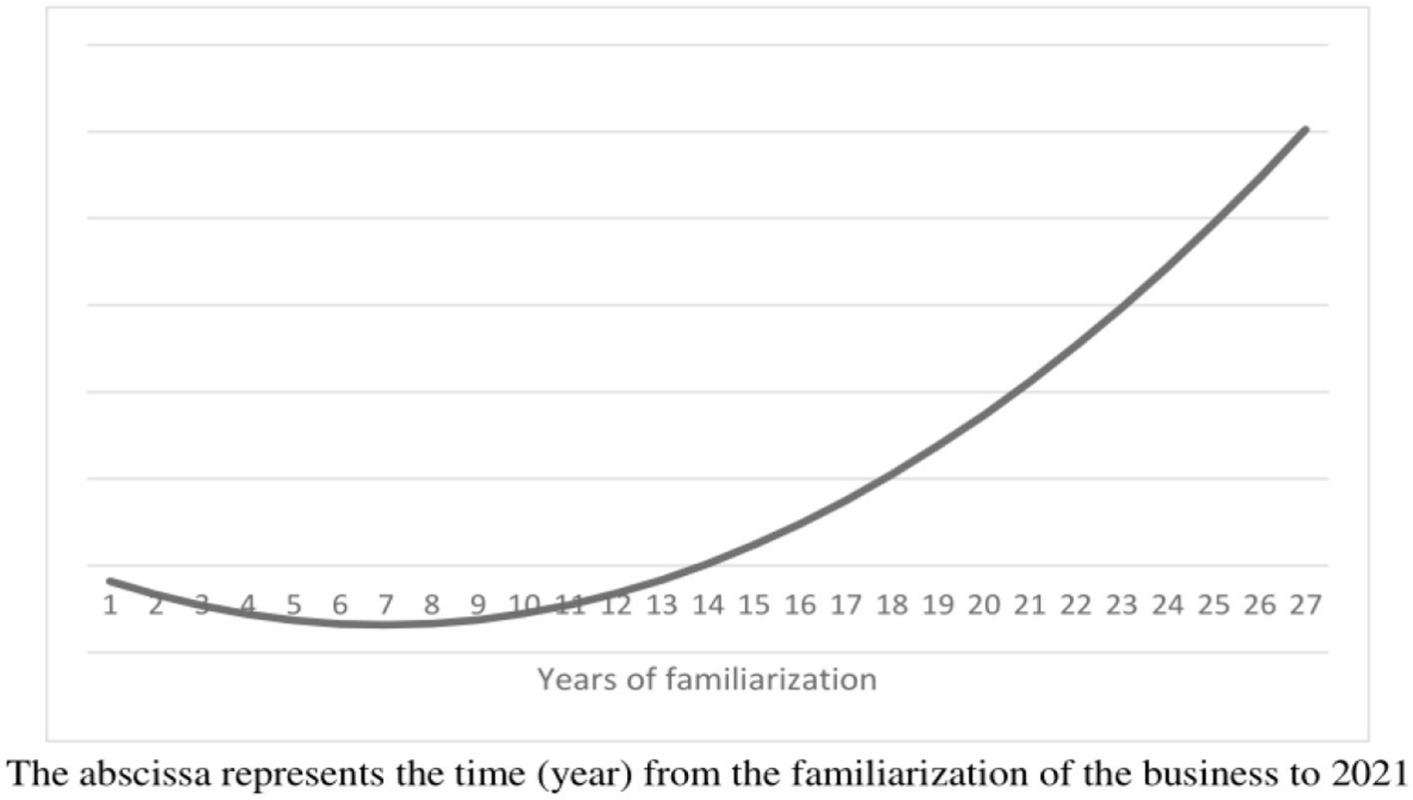
Changes over time in the family business's responsibility for employee health and safety.

As shown in the Tables 6, 7, as the operating time extends, family businesses begin to pay more attention to the health and safety of employees and improve their vocational training. After controlling for many variables, this result still holds true. At the same time, after the quadratic term was introduced, it is found that in the early stage of operation, family businesses tend to be more and more irresponsible for the health and safety of their employees, and once a certain period of time is exceeded, family businesses grow to be more responsible gradually. This shows that the two effects in the H3 play different roles in different periods. Combined with the mechanisms analyzed in this paper, the reason may be that in the first few years of family business operation, in order to make up for its small size, family businesses tend to concentrate on accumulating capital, and then gradually focus on responsibility as they gain a firm foothold. As a whole, this process presents a “U”-shaped change over time, as shown in the Figure 4. Based on the sample calculations here, it can known that this turning point generally occurs in the seventh (6.95) year of family business operations. Out of the 800 family business samples obtained here, 263 were in the first stage, while 537 were in the second stage.

**Table 6:** Regression results of model 1–4.

	**Model 1**	**Model 2**	**Model 3**	**Model 4**
*Fam*_*time*	0.751^***^ (4.88)	0.314^*^ (1.84)	0.966^**^ (2.78)	0.136 (0.34)
**Control variables**	*N*		*N*	
Size		5.916^***^ (5.76)		9.163^***^ (4.50)
Lev		−0.642 (−0.98)		4.873^***^ (2.60)
Rate		−5.20 (−0.48)		−2.40 (−0.08)
Number		0.339 (0.80)		1.23 (0.99)
Industry fixed effect	Y	Y	Y	Y
Robust standard error	Y	Y	Y	Y
R-squared	0.150	0.267	0.133	0.180

*Model 1–2 is the regression result of model (7), and model 3–4 is the regression result of model (8)*.

*** *Significant at the 1% level*.

** *Significant at the 5% level*.

* *Significant at the 10% level*.

**Table 7:** Regression results of model 5–6.

	**Model 5**	**Model 6**
*Fam*_*time*	−0.987^***^ (−2.74)	0.837 (0.82)
*Fam*_*time*^2^	0.071^***^ (3.53)	−0.038 (−0.82)
**Control variables**		
Size	6.132^***^ (5.95)	9.047^***^ (4.43)
Lev	−0.591 (−0.92)	4.846^***^ (2.58)
Rate	−7.948 (−0.75)	−0.934 (−0.03)
Number	0.279 (0.68)	1.265 (1.02)
Industry fixed effect	Y	Y
Robust standard error	Y	Y
R-squared	0.286	0.181

*Model 5 is the regression result of model (9), and model 6 is the regression result of model (10)*.

*** *Significant at the 1% level*.

But at the same time, it is also noticed that on the issue of releasing responsibility signals to the outside world, what is not found is that it has changed significantly over time, which shows that H2 is correct from one aspect.

### Robustness

The criteria for determining family businesses from the broad definition of the CSMAR database was replaced with the proportion of the actual controller of the enterprise with actual control over the enterprise. The higher the proportion, the closer the enterprise is to the behavioral pattern of a family business. Companies whose actual controllers have more than 40% of the actual control of the company as family businesses (as shown in Table 4, the mean value of the variable power is about 40%) are identified, and models 7–10 were build up on this basis and the robustness of models 1–4 was re-verified. Table 8 presents the regression results.

**Table 8:** Regression results of model 7–10.

	**Model 7**	**Model 8**	**Model 9**	**Model 10**
*Power*	1.04^***^ (3.28)	0.885^**^ (2.56)	1.359^**^ (2.33)	0.659 (1.00)
Control variables	N	Y	N	Y
Industry fixed effect	Y	Y	Y	Y
Robust standard error	Y	Y	Y	Y
R-squared	0.177	0.232	0.172	0.224

*** *Significant at the 1% level*.

** *Significant at the 5% level*.

The regression results in Table 8 are basically consistent with those in Table 6, indicating that our conclusions are robust to a certain extent.

Models 11–12 were set up to verify the robustness of models 5–6. Table 9 presents the regression results.

**Table 9:** Regression results of model 9–10.

	**Model 11**	**Model 12**
Fam_time	−1.055 (−1.50)	2.455 (1.51)
Fam_time^2^	0.144^**^ (2.34)	−0.133 (−1.33)
Control variables	Y	Y
Industry fixed effect	Y	Y
Robust standard error	Y	Y
R-squared	0.270	0.228

** *Significant at the 5% level*.

The quadratic term of model 11 is significant, which shows that the assumption of U-curve in H4 is robust, which proves the robustness of model 5.

## Discussion

### Evidence from existing research

Family businesses are the most common mode of operation for private Chinese companies, providing a large number of employment opportunities in China. Yet at the same time, as is the case in most developing countries, Chinese family businesses are characterized by their small scale and difficult access to internal information. It also makes scarce the number of studies that focus on the health and safety of its employees. However, it is precisely because of this that the health and safety issues that have been neglected for these employees for a long period of time should also receive more attention. Family businesses are important players that cannot be ignored in the course of achieving sustainable development and building a healthy business environment in China. If the family business does not fulfill its employee health and safety responsibilities satisfactorily, this goal will not be achieved.

In contrast to other CSR research, this study analyzes the behavior of family enterprises by distinguishing the actual responsibility performance behavior of family enterprises from their externally declared responsibility performance behaviors. It also concludes that the actual responsibility performance of the family business is inconsistent with its declared responsibility performance. This conclusion is not contradictory within the framework of our analysis, and was supported by a considerable number of studies. These studies mainly support conclusions drawn here in this paper in following two aspects.

On the one hand, if the company sends a signal of good social responsibility to the outside world and is accepted and believed by external stakeholders, it will bring “social emotional wealth” to the company. For example, as Magnanelli and Izzo (21) point out, an increasing number of banks and lenders are evaluating CSR performance. Du et al. (22) also emphasized that companies with good responsibility performance tend to obtain better conditions in banks and can obtain lower debt costs. In this sense, “socio-emotional wealth” brings tangible wealth to the business. Reputation and image are the two basic elements of a family business (23). Du et al. (24) analyze Chinese firms, showing that internationalization is positively related to corporate philanthropy, this phenomenon means that the responsible companies will be more favored by international clients. Either from the perspective of “socio-emotional wealth” or from the perspective of commercial wealth brought by “socio-emotional wealth,” family businesses are more motivated to acquire “socio-emotional wealth.” Therefore, many studies based on the information provided by family businesses have concluded that family businesses are more willing to be responsible (25).

This conclusion ignores the possibility that the information provided by businesses does not necessarily reflect the reality. This information may be selectively provided by the enterprise or even fake. This is also supported by a large body of literature, for example, Campopiano and De Massis (26) showed that family-owned businesses tend to be less compliant with disclosure; Biswas et al. (27) claimed that family management has a negative impact on corporate social responsibility-related disclosure; Cabeza-García et al. (28) also demonstrated the same from the perspective of family members serving as board chairs. Meanwhile, for social responsibility information, family enterprises are more sensitive to media exposure than non-family enterprises (29). This behavioral pattern of family businesses undoubtedly coincides with the conclusions drawn from our analysis of the principal-agent theory.

The research framework in this thesis was supported by empirical results based on a comprehensive consideration of these factors. At the same time, there are a smaller number of studies on the changes in family enterprises' responsibility performance over time, so the long-term dynamic changes of family enterprises' behaviors toward employees' health and safety responsibility were studied. After a few years of the start-up period, such a company will gradually be willing to take responsibility for the health and safety of employees, facilitating a better understanding of the behavior of family businesses. Some literatures also provide evidence for this. Combs et al. (30)'s research on the top 500 family businesses of S & P shows that these powerful and long-standing family businesses will perform their social responsibilities better than non-family businesses. This also provides us with the prospect of a family business actively fulfilling its responsibility for the health and safety of its employees.

### Limitations

First, due to the availability of information and the cost of acquisition, only cross-sectional data for 1 year were collected. At the same time, limited by the sample size, we cannot introduce too many control variables in order to avoid losing valuable observations as much as possible. Second, only the listed companies from the sample were included, and as for what is the difference between unlisted family businesses and listed family businesses in the area of employee health and safety responsibilities, this will be another question worthy of study. Finally, due to space constraints, the impact of regional differences on the performance of employees' health and safety responsibilities in family enterprises has not been studied. These limitations are expected to be addressed in future study or after we get more available data.

## Conclusions

This study applied the latest relevant data provided by a third-party CSR rating agency to extract proxy variables and also the 2021 Chinese A-share listed companies as a sample to study the current status of Chinese family enterprises in fulfilling employee health and safety. The dimensions of the study include the actual actions of the family business, external propaganda of the family business, and long-term behavior of the enterprise in fulfilling its responsibilities. Compared with other businesses, Chinese family businesses perform worse in actual actions to fulfill employee health and safety responsibilities. In the external publicity on fulfilling employee health and safety responsibilities, no significant difference between Chinese family businesses and other businesses can be identified. This means that family businesses with poor responsibilities may send false signals to the outside world. In the traditional Chinese cultural environment, family businesses as a whole will be more responsible for the health and safety of their employees as their business practices enjoy a longer period of time. As further research proves, Chinese family enterprises' responsibility for employee health and safety is affected by a variety of mechanisms with changes in their operating time. Responsibility performance shows a U-shaped change over time. The turning point of the U-shaped change occurred in the fifth year of family business familiarization.

Combining the contents of the three aspects, the factors that affect the family business' fulfillment of employee health and safety responsibilities can be also sorted out, which could be applied to put forward relevant suggestions to improve the welfare of family business employees. From the perspective of external stakeholders, especially those who can play a supervisory role, such as the government or regulatory agencies in related industries. External stakeholders can require more transparency and information disclosure to improve family enterprises' enthusiasm to fulfill their responsibilities.

## Data availability statement

The original contributions presented in the study are included in the article/supplementary material, further inquiries can be directed to the corresponding author/s.

## Author contributions

All authors listed have made a substantial, direct, and intellectual contribution to the work and approved it for publication.

## Funding

Thanks the support of Beijing Social Science Fund, China, Major Project (21LLLJA075) and Interdisciplinary Construction Project of Beijing Technology and Business University.

## Conflict of interest

The authors declare that the research was conducted in the absence of any commercial or financial relationships that could be construed as a potential conflict of interest.

## Publisher's note

All claims expressed in this article are solely those of the authors and do not necessarily represent those of their affiliated organizations, or those of the publisher, the editors and the reviewers. Any product that may be evaluated in this article, or claim that may be made by its manufacturer, is not guaranteed or endorsed by the publisher.

## References

[1] Zeng T. Corporate social responsibility (CSR) in Canadian family firms. Soc Responsibil J. (2020) 17:703–18. 10.1108/SRJ-12-2019-0410

[2] Prencipe A Bar-Yosef S Dekker HC. Accounting research in family firms: theoretical and empirical challenges. Eur Acc Rev. (2014) 23:361–85. 10.1080/09638180.2014.895621

[3] Berrone P Cruz C Gomez-Mejia LR. Socioemotional wealth in family firms. Fam Bus Rev. (2012) 25:258–79. 10.1177/089448651143535534624035

[4] Margolis JD Walsh JP. Misery loves companies: rethinking social initiatives by business. Administr Sci Q. (2016) 48:268–305. 10.2307/3556659

[5] Jiang G Lee CMC Yue H. Tunneling through intercorporate loans: the China experience. J Financ Econ. (2010) 98:1–20. 10.1016/j.jfineco.2010.05.002

[6] Bertrand M Schoar A. The role of family in family firms. J Econ Perspect. (2006) 20:73–96. 10.1257/jep.20.2.73

[7] Abdullah SN Mohamad NR Mokhtar MZ. Board independence, ownership and CSR of Malaysian large firms. Corp Ownersh Control. (2011) 8:467–83. 10.22495/cocv8i2c4p5

[8] Zellweger TM Nason RS Nordqvist M Brush CG. Why do family firms strive for nonfinancial goals? An organizational identity perspective. Entrepren Theory Pract. (2013) 37:229–48. 10.1111/j.1540-6520.2011.00466.x

[9] Jiang DS Kellermanns FW Munyon TP Morris ML. More than meets the eye: a review and future directions for the social psychology of socioemotional wealth. Fam Bus Rev. (2017) 31:125–57. 10.1177/0894486517736959

[10] Chen L Chen H. The clan involvement, the socio-emotional wealth and the corporate charitable contributions: a case study based on the survey of the private enterprises all over China. Manage World. (2014) 90–101. 10.19744/j.cnki.11-1235/f.2014.08.009

[11] Preuss L Lenssen G. Tax avoidance and corporate social responsibility: you can't do both, or can you? Corp Govern. (2010) 10:365–74. 10.1108/14720701011069605

[12] Prior D Surroca J Tribó JA. are socially responsible managers really ethical? Exploring the relationship between earnings management and corporate social responsibility. Corp Govern. (2008) 16:160–77. 10.1111/j.1467-8683.2008.00678.x

[13] Nekhili M Nagati H Chtioui T Rebolledo D. Corporate social responsibility disclosure and market value: family versus non-family firms. J Bus Res. (2017) 77:41–52. 10.1016/j.jbusres.2017.04.001

[14] Izzo MF Ciaburri M. Why do they do that? Motives and dimensions of family firms' CSR engagement. Soc Responsib J. (2018) 14:633–50. 10.1108/SRJ-08-2017-0148

[15] Wang M. The research based on the game theory of corporate social responsibility mechanism. J Indus Technol Econ. (2013) 33:13–8. 10.3969/j.issn.1004-910X.2013.02.002

[16] Li X Ma J He X Yuan Y. The modern transformation of family governance: co-evolve of family involvement and family formal institution. Nankai Bus Rev. (2018) 21:101–15. Available online at: https://t.cnki.net/kcms/detail?v=ktJVTzZE33TfW_T38dVfSXw-hscUIcXltutvgkg1ZXtM2ZtZnIhhProzyDVrLZCiY4qU7KjiCXcJWiPHeeUfF5tu7gcO3womisvCGGcgyNtOcytef6K1ECm1S5VKy83a&uniplatform=NZKPT

[17] Chen Z Min Y. The impact of family control on corporate social responsibility: from the perspective of socioemotional-wealth theory. Econ Manage J. (2015) 37:42–50. 10.19616/j.cnki.bmj.2015.04.007

[18] Ye Y Li K Hu G. The selective participation in corporate social responsibility in family enterprises. J Beijing Instit Technol. (2019) 21:76–85. 10.15918/j.jbitss1009-3370.2019.1499

[19] Yang Z Ma G Chen J. Entrepreneurs' comprehensive status, family involvement and corporate social responsibility: micro evidence from the survey of private enterprises in China. Econ Perspect. (2021) 101–15. Available onlline at: https://kns.cnki.net/kcms/detail/detail.aspx?dbcode=CJFD&dbname=CJFDLAST2021&filename=JJXD202108007&uniplatform=NZKPT&v=nx2UNGUWnoXXEKY8gtPPLQZUiIHmh-e_RAQzIL8ZJgYt7Hzq3JkK9oYf7C87CaTx

[20] Freitas AC Silva SA Santos CM. Safety training transfer: the roles of coworkers, supervisors, safety professionals, and felt responsibility. J Occup Health Psychol. (2019) 24:92–107. 10.1037/ocp0000125 30024186

[21] Magnanelli BS Izzo MF. Corporate social performance and cost of debt: the relationship. Soc Responsib J. (2017) 13:250–65. 10.1108/SRJ-06-2016-010334791632

[22] Du X Weng J Zeng Q Chang Y Pei H. Do lenders applaud corporate environmental performance? Evidence from Chinese private-owned firms. J Bus Ethics. (2015) 143:179–207. 10.1007/s10551-015-2758-2

[23] Sageder M Mitter C Feldbauer-Durstmüller B. Image and reputation of family firms: a systematic literature review of the state of research. Rev Manage Sci. (2016) 12:335–77. 10.1007/s11846-016-0216-x

[24] Du X Zeng Q Chang Y. To be philanthropic when being international: Evidence from Chinese family firms. J Manage Organ. (2018) 24:424–49. 10.1017/jmo.2017.9

[25] Dawson A Ginesti G Sciascia S. Family-related antecedents of business legality: an empirical investigation among Italian family owned SMEs. J Fam Bus Strat. (2020) 11:100284. 10.1016/j.jfbs.2019.04.003

[26] Campopiano G De Massis A. Corporate social responsibility reporting: a content analysis in family and non-family firms. J Bus Ethics. (2015) 129:511–34. 10.1007/s10551-014-2174-z

[27] Biswas PK Roberts H Whiting RH. The impact of family vs. non-family governance contingencies on CSR reporting in Bangladesh. Manage Decis. (2019) 57:2758–81. 10.1108/MD-11-2017-1072

[28] Cabeza-García L Sacristán-Navarro M Gómez-Ansón S. Family involvement and corporate social responsibility disclosure. J Fam Bus Strat. (2017) 8:109–22. 10.1016/j.jfbs.2017.04.002

[29] Gavana G Gottardo P Moisello AM. Sustainability reporting in family firms: a panel data analysis. Sustainability. (2016) 9:38–56. 10.3390/su9010038

[30] Combs JG Jaskiewicz P Ravi R Walls JL. More bang for their buck: why (and when) family firms better leverage corporate social responsibility. J Manage. (2022). 10.1177/01492063211066057

